# Interethnic Differences in Muscle, Liver and Abdominal Fat Partitioning in Obese Adolescents

**DOI:** 10.1371/journal.pone.0000569

**Published:** 2007-06-27

**Authors:** David Liska, Sylvie Dufour, Tosca L. Zern, Sara Taksali, Anna M.G. Calí, James Dziura, Gerald I. Shulman, Bridget M. Pierpont, Sonia Caprio

**Affiliations:** Department of Pediatrics, Internal Medicine, the Howard Hughes Institute, and the General Clinical Research Center of the Yale University School of Medicine, New Haven, Connecticut, United States of America; Mayo College of Medicine, United States of America

## Abstract

The prevalence of insulin resistance and type 2 diabetes (T2D) in obese youth is rapidly increasing, especially in Hispanics and African Americans compared to Caucasians. Insulin resistance is known to be associated with increases in intramyocellular (IMCL) and hepatic fat content. We determined if there are ethnic differences in IMCL and hepatic fat content in a multiethnic cohort of 55 obese adolescents. We used ^1^H magnetic resonance spectroscopy (MRS) to quantify IMCL levels in the soleus muscle, oral glucose tolerance testing to estimate insulin sensitivity, magnetic resonance imaging (MRI) to measure abdominal fat distribution. Liver fat content was measured by fast–MRI. Despite similar age and % total body fat among the groups, IMCL was significantly higher in the Hispanics (1.71% [1.43%, 2.0%]) than in the African-Americans (1.04% [0.75%, 1.34%], p = 0.013) and the Caucasians (1.2% [0.94%, 1.5%], p = 0.04). Liver fat content was undetectable in the African Americans whereas it was two fold higher than normal in both Caucasians and Hispanics. Visceral fat was significantly lower in African Americans (41.5 cm^2^ [34.6, 49.6]) and was similar in Caucasians (65.2 cm^2^ [55.9, 76.0]) and Hispanics (70.5 cm^2^ [59.9, 83.1]). In a multiple regression analysis, we found that ethnicity independent of age, gender and % body fat accounts for 10% of the difference in IMCL. Our study indicates that obese Hispanic adolescents have a greater IMCL lipid content than both Caucasians and African Americans, of comparable weight, age and gender. Excessive accumulation of fat in the liver was found in both Caucasian and Hispanic groups as opposed to virtually undetectable levels in the African Americans. Thus, irrespective of obesity, there seem to be some clear ethnic differences in the amount of lipid accumulated in skeletal muscle, liver and abdominal cavity.

## Introduction

In African-American and Hispanic youth, T2DM is more common than in individuals of Caucasian origin [1]. The ethnicity-related higher prevalence of T2DM is attributed to a greater degree of obesity and severity of insulin resistance[2]. Indeed, several studies have demonstrated decreased insulin sensitivity and hyperinsulinemia in healthy African-American and Hispanic children compared with their Caucasian peers [3], [4], [5]. The reasons for these ethnic differences have not yet been elucidated.

Hispanic Americans are the second largest and fastest growing ethnic group in the US. Current data on immigration trends to the US indicate that by the year 2050 over 25% of the population will be represented mainly by Hispanics. Hence, in the near future we will witness an increase in the prevalence of diabetes in the US population of much larger proportion than what has been observed thus far [6].

Insulin resistance plays a major role in the pathogenesis of type 2 diabetes and is strongly linked to increased intracellular fat content in both skeletal muscle and liver [7], [8]. More specifically, intracellular accumulation of diacylglycerols (DAG), is increasingly recognized as a possible mediator of alteration in insulin signaling in both skeletal muscle [7] and liver tissues [8], [9]. Both skeletal muscle tissues and the liver are major loci of insulin resistance in obesity. The overall goal of this study was to determine whether the lipid partitioning in skeletal muscle tissue and liver differ in obese African-American, Hispanic and Caucasian youth, of similar age, and % total body fat. ^1^H-MRS was used to quantify non-invasively the intra and extra myocellular (IMCL and EMCL) lipid content of the soleus muscle and fast-gradient echo magnetic resonance pulse sequences (fast-MRI), enabled the measurement of intra-hepatic fat accumulation in a single breath-hold. MRI allowed the assessment of abdominal fat distribution. In addition, insulin sensitivity was estimated using the oral glucose tolerance tests (OGTT) in all subjects and related to intramyocellular, intrahepatic fat content and visceral fat in obese adolescents of different ethnic groups.

## Methods

### Participants

We studied three groups of overweight and obese subjects: 21 Caucasian, 17 African-American and 17 Hispanic adolescents (total n = 55), recruited from The Pediatric Obesity Clinic at Yale University. To be eligible for this study, subjects had to be overweight or obese, (BMI>85^th^ or 95^th^ age and gender specific), to be taking no medications that can alter glucose metabolism, and to be otherwise healthy. In all participants we did a complete physical examination and took a detailed medical history. Ethnicity was determined by self-report. Subjects were asked two separate questions–one on ethnicity and one on race. The ethnicity information was collected first followed by the question on race. Subjects were provided with the option to select more than one racial category. The study was approved by the Human Investigational Committee of the Yale School of Medicine. Written informed consent was obtained from the parents, and written assent was given by the participants.

### Metabolic Studies

#### Oral Glucose Tolerance Test (OGTT)

All subjects were invited to the Yale General Clinical Research Center for an OGTT at 8 a.m. following an overnight fast, as previously reported [10]. The subjects were instructed to consume a diet containing at least 250 g of carbohydrates the day prior to the study and to refrain from vigorous physical activity. Baseline blood samples were obtained from subjects while they were fasting, with the use of an indwelling venous line for measurement of levels of glucose, insulin, lipid profile, free fatty acids (FFAs), adiponectin and leptin. A standard 3-hr OGTT was then performed with the administration of 1.75 g of glucose per kg body weight (maximum dose: 75 g), and blood samples were obtained every 30 minutes for the measurement of plasma glucose, insulin and c-peptide [10].

### Imaging Studies

#### ^1^H-NMR Spectroscopy: Intramyocellular Triglyceride Content

Muscle TG content was measured using a 4.0T Biospec system (Bruker Biospin MRI GmbH, Ettlingen, Germany) using the following protocol: subjects were positioned supine with the calf muscle of their right leg placed within a coil assembly consisting of two ^1^H-surface coils (13 cm diameter) arranged spatially to generate a quadrature field. Once inserted into the isocenter of the magnet, the probe was tuned and matched, and scout images of the lower leg were obtained to ensure correct positioning of the subject and to define a volume of interest within the soleus muscle. Localized shimming was performed using the FASTMAP procedure [11] and typical ^1^H linewidths within the volume of interest were ∼14Hz. Following pulse calibration, localized proton spectra were acquired from a 10×10×10 mm^3^ voxel using a STEAM sequence (excitation pulse 2ms (SLR90), TR 2.5s, TE 15ms, TM 15ms 2500Hz, #2048 points over 2500Hz), with 3 modules of CHESS water suppression. To prevent voxel mis-registration due to chemical shift effects, total lipid content was estimated from the comparison of two spectra: a water-suppressed lipid spectrum (128 scans) and a lipid-suppressed water spectrum (64 scans), with the appropriate peak for each spectrum on-resonance.

^1^H free induction decays (FIDs) were processed using XWINNMR version 6.5 (Bruker Biospin, Germany); FIDs were zero-filled to 32 k points and multiplied by a Lorentzian/Gaussian function (lb-2/gb 0.15) prior to Fourier transformation. After phase and baseline correction, the resonances of each spectrum were fitted using the Nuts-PPC Software package (Acorn NMR, Inc., CA, USA). The lipid spectrum was deconvoluted by fitting up to 8 resonances over the region from 1.5 to 0.8 ppm using the creatine signal at 3.0 ppm as reference. The areas of the EMCL, IMCL and water resonances were corrected for T_1_ and T_2_ relaxation. EMCL and IMCL content was expressed as a percentage of the water content.

#### Fast-MRI: Liver Fat Content

Measurement of hepatic fat accumulation was performed using MRI along with the Dixon method as modified by Fishbein *et al* [12]. The method is based on phase-shift imaging where hepatic fat fraction (HFF) is calculated from the signal difference between the vectors resulting from in-phase and out-of-phase signals. One hepatic slice pair was obtained during a breath-hold of 15 seconds. Using the MRIcro software program, five regions of interest were drawn on each image, and the mean pixel signal intensity level was recorded. The HFF was calculated in duplicate from the mean pixel signal intensity data using the formula: [(Sin-Sout)/(2*Sin)]*100. This technique was introduced at a later stage of the study; therefore, it was performed in a smaller cohort of subjects (8 African-Americans, 8 Hispanics, and 11 Caucasians). In our group the technique of fast–MRI was validated against the ^1^H-MRS of the liver in a group of 20 subjects and we found a strong agreement between the two techniques (r = 0.94, p = 0.001) (personal data from Drs Caprio, Gerald Shulman and Todd Constable).

#### Abdominal MRI: Intra-abdominal and subcutaneous fat depot

Abdominal MRI studies were performed on a Siemens Sonata 1.5-Tesla system. The pulse sequence was a T1-weighted Fast Low Angle Shot Gradent Echo (FLASH). Slices were acquired using a 400cm field of view (TE:4.76, TR:100, 4 excitations, 90° flip angle, matrix: 256×128, bandwidth:140). The mid-axial section was positioned to pass through the L4/L5 disc space. Images were imported into the Yale Bioimage Suite software package [13]. Visceral, subcutaneous, deep subcutaneous and superficial subcutaneous fat areas were determined from the mid-axial slice. The fascia superficialis was used as the division between the deep and superficial subcutaneous fat. Thresholding was applied to separate fat from soft tissue.

#### DEXA

Total body composition was measured by dual-energy X-ray absorptiometry with a Hologic scanner (Boston, MA, USA).

### Analytical Procedures and Calculations

Plasma glucose levels were measured using the YSI 2700 STAT Analyzer (Yellow Springs Instruments), and lipid levels were measured using an Autoanalyzer (model 747-200, Roche-Hitachi). Plasma adiponectin levels were measured by a double antibody-antibody RIA assay from Linco by our research laboratory. The intra- and inter-assay coefficients of variation are 7.1% and 9.5%, respectively. Plasma insulin and leptin levels were measured using an RIA assay from Linco (insulin intra- and inter-assay coefficients of variation: 6.8% and 11.6%, respectively; leptin intra- and inter-assay coefficients of variation: 6.5% and 8.0%, respectively). Estimated insulin sensitivity was calculated using the Matsuda index (whole-body insulin sensitivity index (WBISI), which has been validated by comparison with hyperinsulinemic-euglycemic clamp studies in obese children and adolescents [14]–[15].

### Statistical Analysis

Data are represented as mean±SD, SEM, or 95% confidence intervals, as appropriate. Parameters that were not normally distributed were log-transformed for analysis. Multiple pair-wise comparisons of subjects were performed using ANOVA with post-hoc Bonferroni correction for multiple comparisons between pairs. Adjustment of comparisons for potential confounders was performed using analysis of covariance with main effects for age, sex, percent fat and other relevant covariates where appropriate. Pearson correlation analysis was used when applicable to examine bivariate relationships. To examine the independent association between IMCL, visceral fat, and ethnicity we used multiple regression analysis. A p-value of <0.05 was considered statistically significant. All analyses were performed using SPSS 14.0 for Windows.

## Results

### Anthropometric Characteristics of the Cohort (Table 1)

As reported in Table 1, in the entire cohort, the distribution of sex was not significantly different across ethnicity (p = 1.00). The three groups were similar with regard to age. Weight and height, BMI and BMI z-score were not significantly different among the three groups. Moreover, assessment of whole body composition by DEXA, revealed no significant differences in percent fat, lean body mass, and total body fat mass among the three groups.

**Table 1:** Anthropometric Characteristics (mean±SD)

	*Caucasian*	*African-American*	*Hispanic*
**N**	21	17	17
**Gender (M/F)**	11/10	6/11	11/6
**Age (yrs)**	14.6±1.84	14.7±2.73	15.2±2.4
**Weight (kg)**	100.2±16.6	103.0±19.7	103.0±22.0
**Height (cm)**	168.0±9.8	165.2±12.2	166.4±10.0
**BMI (kg/m^2^)**	35.7±5.72	37.4±5.5	37.0±6.0
**BMIz**	2.30±0.27	2.4±0.24	2.4±0.35
**%Fat**	40.3±6.55	41.2±5.8	38.74±5.53
**Total Fat Mass (kg)**	39.4±12.0	40.37±10.5	39.04±11.0
**Lean Body Mass (kg)**	57.0±9.42	59.2±11.8	59.45±14.6

### Metabolic Profile of the Cohort (Table 2)

The metabolic profile of the participants was in most aspects very similar across ethnicity (Table 2). After adjusting for age, sex, and percent body fat, there was no significant ethnicity main effect or ethnicity*sex interaction in fasting glucose, two-hour glucose, fasting insulin and the 2 hour insulin levels among the groups. Insulin sensitivity, as measured by WBISI tended to be lower in the Hispanic group, there was however no significant ethnicity main effect or ethnicity*sex interaction in insulin sensitivity. For the lipid profile, total cholesterol and LDL concentrations were similar among the three groups. African Americans tended to have lower triglyceride levels and higher HDL-C than both Caucasians and Hispanics. Leptin and adiponectin levels were similar across ethnicity.

**Table 2:** Metabolic profile of the cohort, adjusted for age, gender and percent body fat.

	*Caucasian*	*African-American*	*Hispanic*
**Fasting Glucose (mg/dl)**			
Mean (95% CI)	90.63 (87.3, 94.0)	91.6 (88.0, 95.4)	92.0 (88.2, 95.8)
**Fasting Insulin (µU/ml)**			
Geometric Mean (95% CI)	30.85 (24.8, 38.3)	28.33 (22.0, 36.7)	35.0 (27.3, 45.0)
**2-hour Glucose (mg/dl)**			
Mean (95% CI)	114.7 (104.7, 124.7)	117.1 (106.0, 128.2)	111.0 (100.5, 122.0)
**2-hour Insulin (µU/ml)**			
Geometric Mean (95% CI)	124.3 (82.7, 187.0)	151.0 (93.2, 244.4)	116.4 (75.6, 179.1)
**WBISI**			
Geometric Mean (95% CI)	1.55 (1.17, 2.06)	1.65 (1.2, 2.3)	1.32 (1.0, 1.83)
**HOMA-IR**			
Geometric Mean (95% CI)	6.8 (5.6, 8.5)	6.34 (5.0, 8.1)	7.9 (6.23,10.0)
**Total Cholesterol (mg/dl)**			
Mean (95% CI)	151.5 (129.0, 174.1)	148.3 (123.0, 174.0)	140.2 (116.0,164.8)
**HDL (mg/dl)**			
Mean (95% CI)	39.0 (33.14, 44.8)	47.13 (40.5, 53.7)	38.3 (32.0, 45.0)
**LDL (mg/dl)**			
Mean (95% CI)	86.0(65.1, 106.2)	82.2 (59.0, 106.0)	81.4 (59.0, 104.0)
**Triglycerides (mg/dl)**			
Geometric Mean (95% CI)	93.4 (65.8, 132.7)	80.2 (53.8, 119.3)	94.4 (64.4, 138.4)
**FFA (µmol/L)**			
Geometric Mean (95% CI)	466.4 (363.2, 598.8)	377.3 (279.0, 510.3)	534.0 (407.1, 701.0)
**Leptin (ng/ml)**			
Geometric Mean (95% CI)	23.3 (20.0, 27.1)	28.0 (24.0, 33.0)	23.0 (19.3, 26.3)
**Adiponectin (µg/ml)**			
Geometric Mean (95% CI)	8.14 (5.6, 11.8)	8.7 (5.5, 13.7)	7.8 (5.2, 12.0)

### Muscle, liver and Abdominal lipid Partitioning (Figure 1)

**Figure 1:**
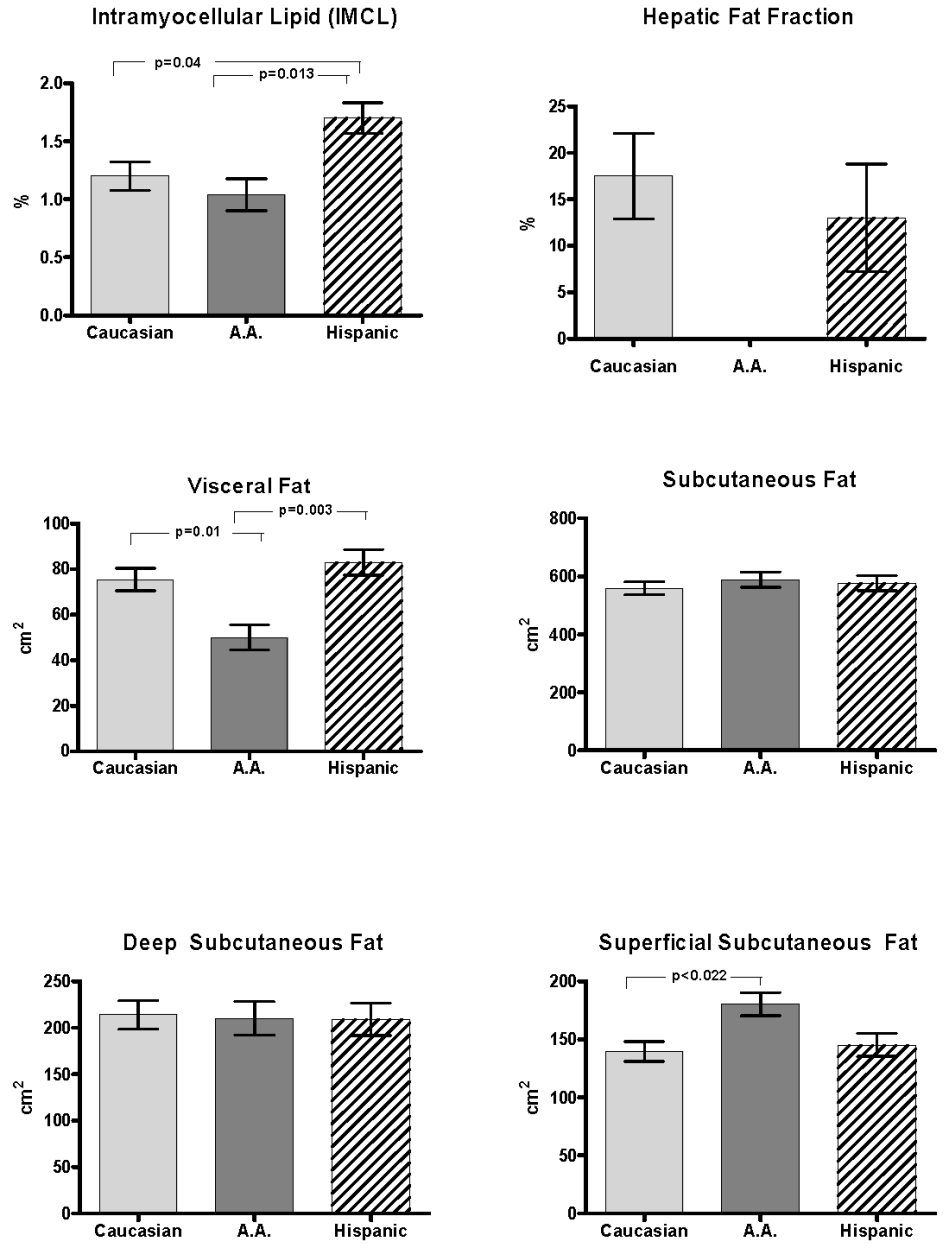
Visceral fat, subcutaneous fat (total, deep, and superficial), intramyocellular lipid (IMCL), and hepatic fat fraction by ethnicity (mean±SEM, adjusted for age, gender, and percent fat).

As shown in Figure 1, despite a similar degree of overall adiposity, IMCL was significantly higher in the Hispanic group (1.71%, 95% CI: 1.43%, 2.0%) than in the African-American (1.04%, 95% CI: 0.75%, 1.34%) and the Caucasian (1.2%, 95% CI: 0.94%, 1.5%) groups (p = 0.013 and p = 0.04 respectively). In contrast, EMCL levels were not significantly different among the Caucasian (1.78%, 95% CI: 1.43%, 2.1%), African-American (1.81%, 95% CI: 1.4%, 2.2%) and Hispanic groups (2.0%, 95% CI: 1.64%, 2.4%) (data not shown).

While intrahepatic fat content, expressed as hepatic fat fraction (HFF) was undetectable in the AA obese adolescents, in the Caucasian HFF it was 17.5% and in the Hispanics 13%. These elevated HFF levels denote increased lipid accumulation in the liver since they are well above the accepted normal reference value of 5.5% [16].

Abdominal visceral fat was significantly lower in the African-American group (49.8 cm^2^, 95% CI: 38.0, 61.7 cm^2^) compared to the Caucasian (75.4 cm^2^, 95% CI: 65.0, 86.0 cm^2^) and Hispanic (83.0 cm^2^, 95% CI: 70.7, 95.0 cm^2^) groups (p = 0.01 and p = 0.003, respectively), after adjusting for age, sex, and percent body fat. Total subcutaneous fat was similar in the African-American group (589.0 cm^2^, 95% CI: 534.0, 644.1 cm^2^) , in the Caucasian group (559.0 cm^2^, 95% CI: 510.3, 607.3 cm^2^) and the Hispanic group (577.0 cm^2^, 95% CI: 521.3, 632.4 cm^2^) . We further divided the subcutaneous fat into deep and superficial and found that there were no significant differences in the deep subcutaneous fat between the three groups. In contrast, the African American group had a significantly higher amount of superficial subcutaneous fat (180.3 cm^2^, 95% CI: 159.0, 202.0 cm^2^) than the Caucasian group (139.5 cm^2^, 95% CI: 121.0, 158.1 cm^2^) but not compared to the Hispanic group (145.2 cm^2^, 95% CI: 124.0, 166.5 cm^2^). The Caucasian and Hispanic groups were similar with respect to all abdominal depots.

**Figure 2:**
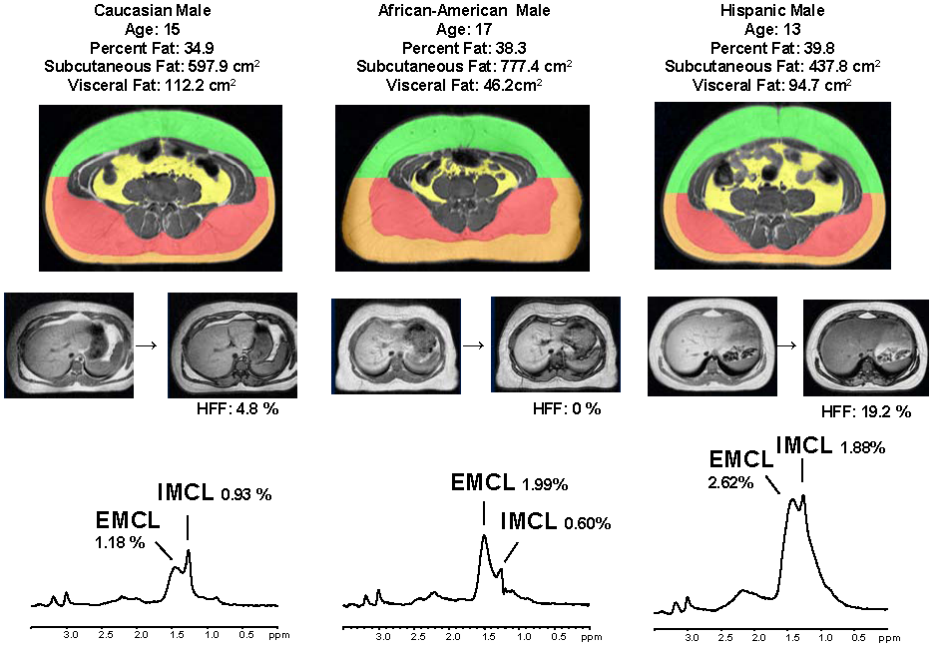
Representative abdominal MRI image, liver scans and ^1^H-MRS of soleus muscle spectra from one Caucasian, one African American, and one Hispanic boy.

To further illustrate the marked ethnic differences in IMCL, HFF and abdominal fat partitioning we chose one boy from each group. As shown in Figure 2, the 3 boys had similar age and % total body fat. Nevertheless, while the Hispanic boy had marked elevation in IMCL, HFF and visceral fat, the AA boy, in contrast had low IMCL, undetectable liver fat and low visceral fat and marked expansion of the total subcutaneous fat. The Caucasian boy had a low IMCL and liver fat but significant visceral fat content.

### Relationships between IMCL, Hepatic Fat fraction, Visceral fat, and Insulin Sensitivity (Figure 3)

**Figure 3:**
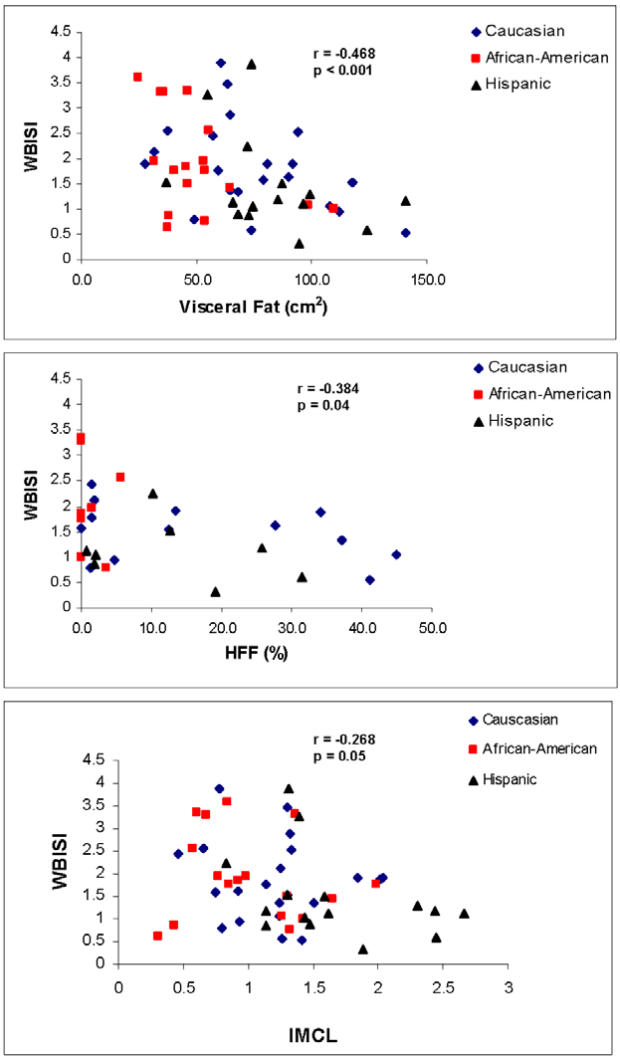
Relationships between IMCL, Hepatic Fat Fraction (HFF), Visceral Fat, and insulin sensitivity (WBISI) in all 3 ethnic groups.

Using univariate analysis in the entire cohort, significant inverse relationships were found between insulin sensitivity (WBISI) and IMCL (r = −0.268; p = 0.05), hepatic fat content (HFF) (r = −0.384, p = 0.04), and with visceral adiposity (r = −0.468; p = 0.001) (Figure 3).

To further analyze to what degree any differences in IMCL and Visceral fat may be accounted for by ethnic differences we used a stepwise multiple regression analysis to determine whether the effect of ethnicity remained after adjusting for confounding variables such as gender, age and % body fat. Of note, ethnicity alone explained 10% of the variance of IMCL (r^2^ = 0.103, p<0.02), and this relationship was not affected by entering age, gender and % body fat to the model. On the other hand, ethnicity alone was not found to significantly account for differences in visceral fat. Indeed, better predictors for visceral fat were gender and % total body fat. In a multiple regression analysis using WBISI as the dependent variable and ethnicity, gender, IMCL, visceral and HFF as independent variables, we found that ethnicity explained 10.4% (p<0.078) and visceral 32.3% (p<0.01) of the variance in insulin sensitivity.

## Discussion

Our study compared IMCL level, determined by ^1^H-MRS and liver fat determined by fast-MRI, across ethnicity in a pediatric population of obese adolescents with normal glucose tolerance. Given the higher incidence of insulin resistance and T2DM in minority populations, we hypothesized that obese Hispanic and African-American youth would have higher IMCL and liver fat content than their Caucasian counterparts. Surprisingly, our study showed that African-Americans had lower IMCL and liver fat content than Hispanics. In contrast, the Hispanic adolescents in our study had significantly higher IMCL levels compared to their Caucasian and African-American peers, and had a significantly greater lipid content in the liver than the African Americans. Our data suggest that obese Hispanic adolescents have a higher propensity to accumulate excess lipid inside the myocyte, in contrast to obese Caucasians and African-Americans. The ethnic differences in IMCL are independent of age, gender and overall adiposity. Moreover, the liver of these obese Hispanic adolescents like that of the Caucasians had a twofold increase in hepatic fat fraction than the AA group. Indeed, a recent population-based study of obese adolescents found that Non Alcoholic Fatty Disease (NAFLD) is more prevalent in Hispanics than in Caucasians, and is the least common in African Americans [17]. It is unclear why Hispanics tend to be more susceptible to liver and IMCL accumulation. Possible causes of these different phenotypes include genetic factors in the metabolic processing and storage of excess fat and/or environmental differences such as diet and exercise. It is very likely that the increased susceptibility of Hispanics to deposit lipid inside the liver and myocyte plays an important role in their increased risk of developing insulin resistance, T2DM, and other metabolic dysfunction. In accordance with the increased IMCL are the higher levels of plasma free fatty acids we found in Hispanics as compared to African Americans. These elevated levels of free fatty acids may lead to an increased flux to the liver and muscle, leading to the formation of lipid moieties such as diacylglycerol and ceramide, which have been shown to alter insulin signaling and thus lead to insulin resistance [7], [8].

Our study reaffirmed that the regional abdominal fat distribution in African-American adolescents is very different from that of their Caucasian and Hispanic peers, of similar adiposity, age and gender. Consistent with other studies [4], [5], [21] the African-American group in our study had significantly lower visceral fat than the Caucasian and Hispanic groups. In addition, we further divided the subcutaneous fat into deep and superficial subcutaneous, using the fascia superficialis as the partition. We found in the African-Americans a greater amount of superficial subcutaneous fat as compared to Caucasians. In line with the lower visceral fat content, we found African Americans to have undetectable levels of intrahepatic fat, which is consistent to the low prevalence rate of fatty liver reported in this ethnic population by Schwimmer *et al* [17]. Thus, in African American obesity seems to spare excessive accumulation of fat not only in the visceral compartment but also in the liver and muscle (IMCL). Of note, low level of visceral fat accumulation and excess of subcutaneous abdominal fat have been reported among individuals of African descents, whether resident in the US, the Caribbean, South America or Europe [18]. The mechanism for the ethnic disparities in fat partitioning has not been elucidated.

It is possible that factors similar to those that protect African-Americans from accumulating visceral fat may also be responsible for their lower liver and IMCL lipid content. Our study suggests that the higher rates of insulin resistance that have been observed in the African-American population cannot be attributed to increased visceral fat or IMCL. Of note, despite their lower visceral fat and lower IMCL levels, African-Americans in our study had a similar degree of insulin resistance to the other groups. It seems clear that the fat compartments we analyzed are not directly responsible for the insulin resistance observed in African-Americans. It is likely that the pathogenesis of insulin resistance and T2DM in African-Americans is linked to a mechanism other than abnormal fat deposition. The low level of both visceral and intrahepatic fat in the African Americans may explain their better lipid profile and the lower prevalence rate of metabolic syndrome seen in this ethnic group [19].

Interestingly, while several studies have shown that African-American and Hispanic adolescents are more insulin resistant than Caucasian adolescents [2]–[5], [21]; in our study insulin sensitivity as measured by the OGTT was not significantly different among the three groups. However, this finding is not surprising considering that our study consisted of a very homogenous obese group. Bacha *et al* [21] made a similar observation when comparing insulin sensitivity between Caucasian and African-American obese adolescents, ascribing the similarity in insulin sensitivity to the overriding effect of obesity-related insulin resistance that masked ethnicity-related differences in insulin sensitivity. The cross-sectional nature of this study does not allow us to deduce a cause-effect relationship. Further limitations are due to the lack of information on physical fitness and composition of their diet. Although the ethnic differences in fat partitioning were readily apparent we believe that our findings need confirmation in larger groups of obese children of different ethnicity.

In conclusion our study suggests that obese Hispanic adolescents have greater IMCL lipid content than both Caucasians and African Americans, with comparable % fat, age and gender. Excessive accumulation of fat in the liver was found in both Caucasian and Hispanic groups as opposed to virtually undetectable levels in the African American obese adolescents. Thus, despite a seemingly overall degree of obesity, there seem to be some distinct ethnic differences in the amount of lipid accumulated in skeletal muscle, liver and abdominal cavity. The increased IMCL and liver fat content in the obese Hispanic adolescents has important implications for future health in this ethnic group, given that both phenotypes are associated with T2DM and steatohepatitis which may progress to more serious hepatic conditions at a very young age. Moreover, our study would suggest that these ethnic differences in tissue lipid partitioning should be considered when designing an intervention study to prevent or treat the associated complications of obesity in youth.
